# Extracorporeal shock wave therapy as a treatment option for persistent clitoral priapism: a case report

**DOI:** 10.1093/sexmed/qfae082

**Published:** 2024-12-03

**Authors:** Karis Buford, Lauren Phung, Bernadette M M Zwaans, Priya Padmanabhan, Rachel S Rubin, Kenneth M Peters

**Affiliations:** Department of Urology, Corewell Health William Beaumont University Hospital, Royal Oak, MI 48073, United States; Oakland University William Beaumont School of Medicine, Rochester, MI 48309, United States; Department of Urology, Corewell Health William Beaumont University Hospital, Royal Oak, MI 48073, United States; Oakland University William Beaumont School of Medicine, Rochester, MI 48309, United States; Department of Urology, Corewell Health William Beaumont University Hospital, Royal Oak, MI 48073, United States; Oakland University William Beaumont School of Medicine, Rochester, MI 48309, United States; Georgetown University Hospital Department of Urology, Washington, DC 20007, United States; Department of Urology, Corewell Health William Beaumont University Hospital, Royal Oak, MI 48073, United States; Oakland University William Beaumont School of Medicine, Rochester, MI 48309, United States

**Keywords:** clitoral priapism, clitoris, clitoral engorgement, Li-ESWT, case report

## Abstract

**Introduction:**

Clitoral priapism is persistent clitoral engorgement without sexual stimulation. Presentation is sparse, and therefore limited treatment options have been investigated.

**Aim:**

We present a case report of a 34-year-old female presenting with persistent nonischemic clitoral priapism 5 years after aggressive clitoral stimulation.

**Methods:**

Patient underwent six weekly Li-ESWT sessions at frequency 4 Hz, energy 0.11 mJ for 2000 shocks per session. Assessment included physical examination of clitoral glans engorgement and retraction, global response assessment (GRA) score, need for topical phenylephrine, and ability to achieve orgasm.

**Results:**

At the end of the therapy, examination revealed complete resolution of priapism with a normal-appearing clitoris fully retracted behind the clitoral hood. The patient reported no longer requiring topical phenylephrine, a significant improvement in GRA, and the ability to achieve orgasm.

**Conclusion:**

We present a case of nonischemic clitoral priapism resolved with Li-ESWT. More investigation regarding the utilization of Li-ESWT in the treatment of clitoral priapism is highly encouraged.

## Introduction

Priapism is defined as a complete or partial sustained erection that lasts for more than 4 h without sexual excitation. ^1^ While penile priapism is well described, clitoral priapism is uncommon with few cases reported and fewer treatment options investigated. This presents a challenge in establishing effective workup and management. Here, we investigate the question: is low-intensity extracorporeal shock wave therapy (Li-ESWT) an effective treatment option for a patient presenting with high-flow clitoral priapism?

## Case presentation

### Patient presentation

A 34-year-old female presented with severe clitoral pain that worsened with light touch, anorgasmia, sexual aversion, and mild urinary hesitancy and urgency. Her symptoms began 5 y prior to presentation after aggressive clitoral stimulation during a consensual sexual encounter. She had no medical history or relevant family medical history, and a surgical history of cosmetic rhinoplasty.

**Figure 1:**
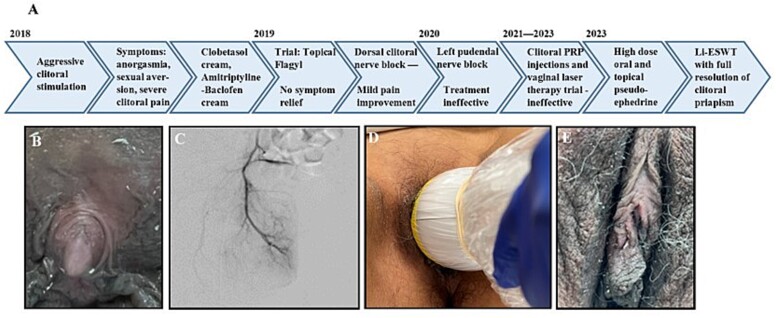
(A) Timeline of prior treatments. (B) Initial examination revealing clitoral engorgement. (C) CT angiogram revealing no vascular abnormality of bilateral internal pudendal arteries. (D) Li-ESWT device (SoftWave®). (E) Clitoris after third Li-ESWT treatment. Note complete resolution of engorgement.

Prior to the presentation, the patient underwent several ineffective therapies by outside providers for presumed chronic clitorodynia. These included: dorsal clitoral nerve block, clitoral adhesion release, pudendal nerve blocks, and clitoral platelet-rich plasma injection. Figure 1A outlines the treatments she received. She eventually received a diagnosis of clitoral priapism and presented for evaluation.

### Diagnostic assessment

Physical examination revealed engorged glans clitoris with complete retraction of the clitoral hood and proximal adherence of prepuce (Figure 1B). Glans engorgement was assessed by the same provider based on the appearance of protrusion beyond the clitoral hood. An unremarkable lumbar magnetic resonance imaging (MRI) ruled out nerve compression, and computed tomography (CT) angiogram ruled out an arteriovenous fistula (Figure 1C). About 30 mg of oral pseudoephedrine provided an unsustained moderate decrease in glans engorgement and topical phenylephrine provided slight symptomatic relief.

### Therapeutic intervention

The patient underwent six weekly Li-ESWT sessions with SoftWave® Model OW100S-US (Revision V.012022.11.23; Manufacturer MTS Science, Germany). The probe was applied to the clitoris (Figure 1D). Therapy was commenced at frequency 4.0 Hz and energy 0.11 mJ for 2000 shocks per session. Qualitative assessment at weekly intervals and validated questionnaires were utilized as no standard assessment for the investigation of clitoral priapism currently exists. Assessment included physical examination of clitoral glans engorgement and clitoral hood retraction, patient-reported pain scores, global response assessment (GRA) score, need for topical phenylephrine, and ability to achieve orgasm.

### Follow-up and outcomes

After the first session, the patient’s pain decreased, with subjective mild improvement of glans engorgement on exam. After the second and third sessions, she was no longer utilizing topical phenylephrine cream with clitoris partially retracted behind the clitoral hood. After the fourth session, patient reported sustained significant improvement of clitoral pain and voiding symptoms. She reported having engaged in sexual stimulation with an associated small orgasm. Examination revealed complete resolution of priapism with a normal-appearing clitoris fully retracted behind the clitoral hood. Following the sixth session, the patient reported she was significantly improved on GRA, self-reporting an 80% improvement in symptoms overall. Six weeks after the final session, the patient reported sustained improvement of clitoral priapism as well as complete resolution of her sexual aversion. She reported an 80% improvement in clitoral pain, with some difficulty achieving orgasm due to discomfort with manual clitoral stimulation. However, she noted the ability to achieve weak orgasms.

### Patient perspective

The patient states that Li-ESWT sessions were easily tolerated with minimal pain and without adverse outcomes. Should she require it, this is an intervention she would undergo again. She expresses hope that her orgasms continue to improve.

## Discussion

The most commonly reported etiologies of clitoral priapism are medications including selective serotonin reuptake inhibitors or tricyclic antidepressants,^2^ along with other causes such as hematologic conditions.^3^ The condition is considered to be similar to penile priapism and is more likely to be due to blockage of cavernosal venous outflow due to alpha-sympathetic blockade promoting smooth muscle relaxation.^3^

Prior to treatment, one should differentiate between ischemic and non-ischemic priapism. While a well-established workup has yet to exist, an ultrasound (US) of the clitoral shaft with greyscale revealing fibrosis and diminutive cavernosal artery color Doppler US (CDU) may assist the provider with the diagnosis of ischemic clitoral priapism.^4^ Nonischemic causes of high arterial blood flow are less common in clitoral priapism as compared to male priapism. If nonischemic priapism is suspected, CDU can be used as an initial imaging test in which the presence of turbulent cavernous blood flow may reveal an arterio-cavernous fistula. A computed tomography angiogram (CTA) may also be utilized—particularly if the source of abnormal blood flow is not identified on CDU.^5^ In a case report of penile high-flow priapism, the source was equivocal on Doppler US, but follow-up CTA revealed arterial enhancement and pooling at the site of arteriovenous fistula.^6^

Our patient had prolonged symptoms without evidence of ischemia and with substantial improvement of clitoral function upon completion of Li-ESWT. Thus, the diagnosis of high flow priapism was given based on history and clinical evaluation. This was likely coupled with an aspect of pelvic floor dysfunction due to levator ani hypertonicity induced by clitoral pain. This may have been the etiology of her voiding symptoms, which resolved as her pain improved.

The constellation of underreporting cases of clitoral priapism and the absence of a concrete diagnostic and treatment protocol pose a challenge in determining effective management. A review of published case reports notes potential treatments include removal of the offending medication, intracavernous injection of α-adrenergic agonists, conservative therapy, and surgical management among others (Table 1).

**Table 1:** Relevant treatment options for clitoral priapism.

**Management strategies**	**Article(s)**
Withdrawal of the offending medication (trazodone, fluoxetine, bromocriptine, bupropion, and citalopram commonly reported)	Yafi et al.^3^; Goldstein^4^
Drainage of cysts with injection of fibrin glue (PGAD from perineural or sacral nerve root cyst)	Yafi et.al.^3^
Transcutaneous electrical nerve stimulation (PGAD from sensory neuropathy of dorsal nerve of the clitoris)	Yafi et.al.^3^
Intracavernosal injection of phenylpropanolamine and phenylephrine with or without aspiration	Yafi et.al.^3^; Goldstein^4^
Cavernosal-spongiosal shunt with snake maneuver	Goldstein^4^
NSAIDs, ice packs, and opiates	Gharahbaghian^2^
Oral pseudoephedrine (administered around the clock for 24 h followed by 48 h of maintenance therapy)	Unger and Walters^7^
Oral pseudoephedrine (administered every 8 h for a total 4 months)	Gürpınar Tosun et al.^8^

Abbreviation: PGAD, persistent genital arousal disorder.

Extracorporeal shock waves represent acoustic energy that causes pressure fluctuations in the receiving cells.^9^ Li-ESWT decreases inflammation, promotes angiogenesis, and recruits stem cells. Several utilizations for Li-ESWT have been implicated, including pain associated with Peyronie’s disease, erectile dysfunction, and pelvic pain.^10^ Li-ESWT’s anti-inflammatory and angiogenic effects make it a feasible therapy for patients presenting with high-flow clitoral priapism. Limitations associated with this case report include the limited nature of the investigation. Future investigations may provide insight into the use of Li-ESWT in the treatment of both clitoral and penile priapism.

## Conclusion

Although the incidence of clitoral priapism is low, its potentially emergent nature, painful symptoms, and impact on patients’ quality of life warrant continuous investigation of potential treatments. Here, we present a patient with sustained resolution of refractory clitoral priapism after Li-ESWT. Future investigation regarding the utilization of Li-ESWT in the management clitoral priapism is needed.
